# Efficiently quantifying DNA methylation for bulk- and single-cell bisulfite data

**DOI:** 10.1093/bioinformatics/btad386

**Published:** 2023-06-16

**Authors:** Jonas Fischer, Marcel H Schulz

**Affiliations:** Department of Biostatistics, Harvard T.H. Chan School of Public Health, Boston, MA 02115, United States; Department for Computational Biology and Applied Algorithmics, Max Planck Institute for Informatics, Saarbrücken 66123, Germany; Department for Computational Biology and Applied Algorithmics, Max Planck Institute for Informatics, Saarbrücken 66123, Germany; Institute of Cardiovascular Regeneration, Department of Medicine, Goethe University, Frankfurt am Main 60590, Germany; Cardio-Pulmonary Institute, Goethe University, Frankfurt am Main, Germany; German Centre for Cardiovascular Research, Partner Site Rhein-Main, Frankfurt am Main 60590, Germany

## Abstract

**Motivation:**

DNA CpG methylation (CpGm) has proven to be a crucial epigenetic factor in the mammalian gene regulatory system. Assessment of DNA CpG methylation values via whole-genome bisulfite sequencing (WGBS) is, however, computationally extremely demanding.

**Results:**

We present FAst MEthylation calling (FAME), the first approach to quantify CpGm values directly from bulk or single-cell WGBS reads without intermediate output files. FAME is very fast but as accurate as standard methods, which first produce BS alignment files before computing CpGm values. We present experiments on bulk and single-cell bisulfite datasets in which we show that data analysis can be significantly sped-up and help addressing the current WGBS analysis bottleneck for large-scale datasets without compromising accuracy.

**Availability and implementation:**

An implementation of FAME is open source and licensed under GPL-3.0 at https://github.com/FischerJo/FAME.

## 1 Introduction

DNA methylation has been shown to be an essential building block of the gene regulatory system (Mattei *et al.* 2022), being often referenced as the “fifth base”. As an epigenetic mark, it has been shown to be crucial for dynamic gene regulation throughout development and aging (Smith and Meissner 2013, Field *et al.* 2018, Bashkeel *et al.* 2019, Greenberg and Bourc’his 2019) and aberrant methylation has been reported in different diseases, especially having a broad impact on cancer (Lister and Ecker 2009, Kulis and Esteller 2010, Huang *et al.* 2015, Papanicolau-Sengos and Aldape 2022, Wang *et al.* 2022). DNA methylation can be measured large scale using methylation arrays (Pidsley *et al.* 2016) or using sequencing (Meissner *et al.* 2005, Krueger *et al.* 2012). Assessment of genome-wide DNA methylation status via bisulfite sequencing (BS) is considered as the gold standard for methylation profiling. The current developments in sequencing technology, however, led to a drastic increase in the amount of data produced, on the one hand due to higher throughput and on the other hand due to the development of single-cell BS approaches in which hundreds of individual cells are analysed (Smallwood *et al.* 2014, Farlik *et al.* 2015, Schwartzman and Tanay 2015, Liu *et al.* 2021).

While there have been many attempts to speed-up the computation of DNA CpG methylation (CpGm) values, all previous approaches have relied on a two-step procedure. Bisulfite reads are first aligned to the genome and then CpGm values are computed from read alignment files.

This separation into two steps creates an unnecessary overhead if the primary interest lies in the CpGm values, which is usually the case for mammalian genomes, as bisulfite alignment files consume large amounts of disk space, imposing extra I/O time and disk resources for the computations. Furthermore, most methods are built around existing read alignment software that was originally developed for traditional DNA read alignment. However, WGBS reads are more difficult to align as read T nucleotides may map to genomic C nucleotides. This not only requires more specialized index structures compared to traditional DNA alignment (Supplementary Fig. S1), but also introduces mapping ambiguity for read T nucleotides, referred to as the asymmetric mapping problem (Lister and Ecker 2009).

**Figure 1.**
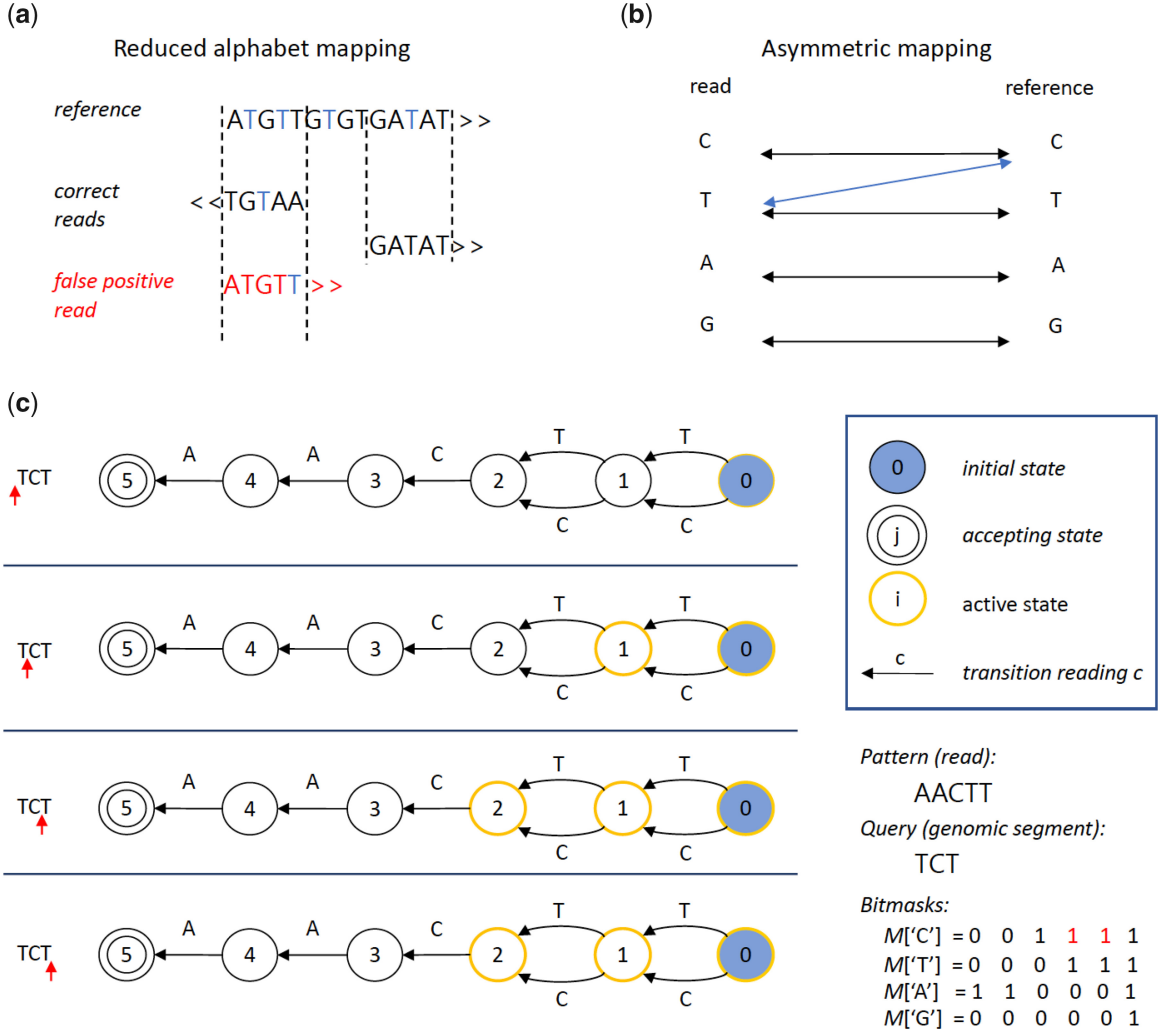
Alignment aware mapping. (a) Errors made through traditional reduced alphabet mapping. Former C reduced to T are highlighted, strand origin indicated by arrows. (b) Principle of asymmetric aware mapping, T can be mapped unidirectionally to C. (c) Example of asymmetric (bisulfite-aware) Shift-And automaton. Visualization of the query of the letter sequence [i.e. genomic segment “TCT” for the pattern “AACTT” (i.e. read)]. The current position in the segment (left side) is indicated by a red arrow.

Based on how the asymmetric mapping problem is handled, available methods for WGBS read alignment can be classified into reduced alphabet (RA) and bisulfite-aware (BA) mapping approaches. RA mappers exchange C residues to T in all sequences (reads and reference), obtaining a reduced three letter alphabet, such that bisulfite converted cytosines do not count as sequencing errors during mapping. This reduction leads to false positive mappings in the genome (Fig. 1a) and, hence, less sensitive methylation calls. Popular RA mappers are Bismark (Krueger and Andrews 2011), BSSeeker (Chen *et al.* 2010, Guo *et al.* 2013), BratNova (Harris *et al.* 2016), as well as gemBS (Merkel *et al.* 2019), and bwameth (Pedersen *et al.* 2014), which use efficient DNA aligners such as Bowtie (Langmead and Salzberg 2012) or bwa (Li and Durbin 2009) to build an index of CtoT and GtoA converted genome sequences.

While widely deployed, RA mappers do not correctly address the asymmetric mapping problem and have been shown to have reduced accuracy (Frith *et al.* 2012, Otto *et al.* 2012). This is because they do not properly account for wrong mapping positions due to the reduced alphabet. In contrast, BA methods use different approaches to resolve the mapping ambiguity: (i) realign potential reads at positions obtained with the reduced alphabet using a BA alignment method (Huang *et al.* 2018), (ii) use a *k*-mer based index with bitmasks to address converted cytosines (Xi and Li 2009), or (iii) directly solve the asymmetric mapping problem with a modified alignment algorithm (Frith *et al.* 2012, Otto *et al.* 2012). BA mappers such as Segemehl (Otto *et al.* 2012) are among the most accurate methods, but are very slow and, hence, hardly scale to the massive amounts of data currently being produced. While there have been many attempts to speed-up the bisulfite alignment process, the extended throughput in current single cell datasets is making analysis even more challenging and customized single cell analysis methods are needed (Wu *et al.* 2019).

Here, we present FAME (Fast and Accurate MEthylation calling), the first BA mapping method with an index that is tailored for the alignment of BS reads with direct computation of CpGm values. Our method is built on a novel data structure that exploits spaced *k*-mer counting within short segments of the genome to quickly reduce the genomic search space. FAME is optimized to handle large single cell datasets. With the resulting index structure, FAME enables ultra-fast and parallel querying of reads without I/O overhead. We evaluated the performance of FAME on both synthetic and real data in comparison to the state-of-the-art tools. All experiments showcase the unique ability of FAME to retrieve as accurate results as the best state-of-the-art methylation caller in just a fraction of time. With an order of magnitude faster processing time and no need to write intermediate alignment files to disk for the final methylation calling, it overcomes the current bottleneck in BS-seq based CpG methylation calling, easily scaling to large single cell processing tasks.

## 2 Approach

To solve bisulfite-aware WGBS mapping efficiently, we propose **F**ast and **A**ccurate **ME**thylation calling (FAME). Our method consists of two main building blocks, an index structure specifically tailored to model CpGm methylations, and an efficient alignment method that implements asymmetric mapping of read Ts to reference Cs based. We next give a more detailed overview of FAME and then discuss the index and alignment separately.

### 2.1 FAME

**Figure 2.**
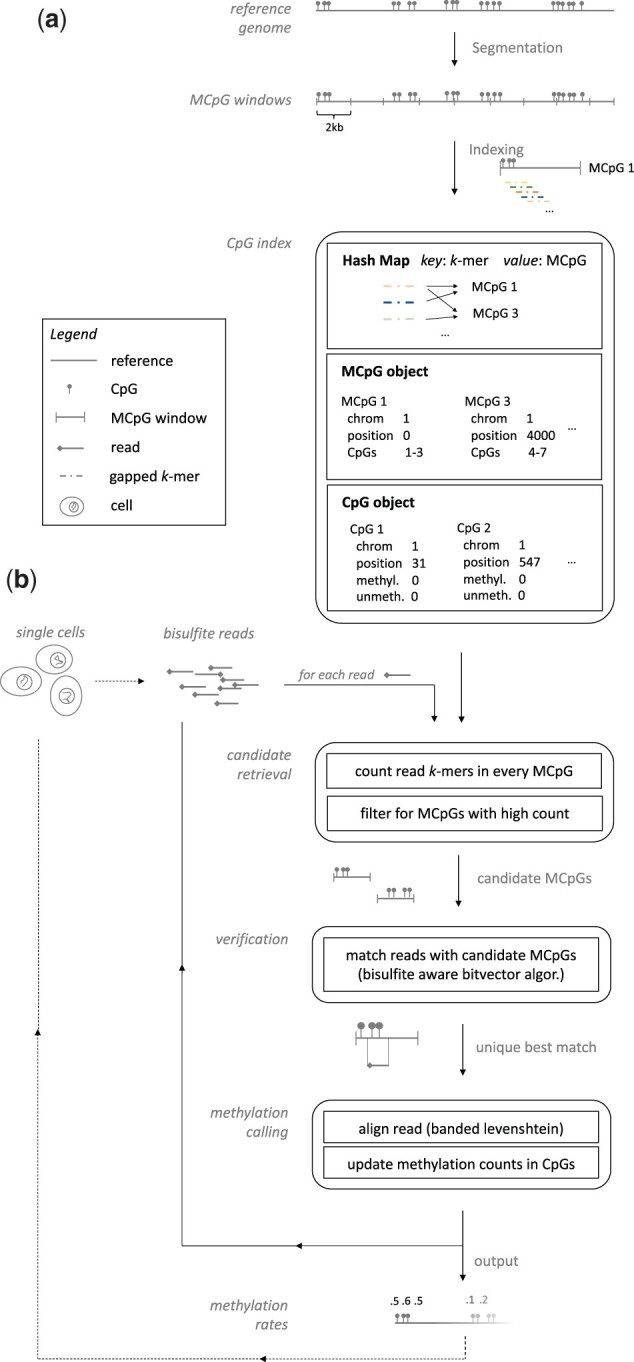
FAME workflow. General workflow of FAME for index construction (a) and read alignment (b). For a given reference genome (top), a CpG index is constructed for 2 kb genomic segments called Meta CpGs (MCpGs) using a rolling hash function for spaced *k*-mers. WGBS reads are matched in three phases (bottom): candidate retrieval, verification, and methylation calling. Methylation rates are updated in a separate data structure, directly yielding methylation values without any re-alignment. FAME can process bulk or single cell datasets.

FAME is a bisulfite aware mapper that is built on top of a custom indexing and alignment method, an overview of which is given in Fig. 2. It leverages a spaced *k*-mer based index structure (Fig. 2a), that enables ultra-fast retrieval of small genomic segments of large genomes, where each of these segments contains a potential match for the given read. We call these genomic segments Meta CpGs, or MCpGs in short, as they resemble a collection of CpGs in our index structure. This index structure is fixed for a given reference genome and thus can be queried in parallel for multiple reads. Furthermore, it contains counting structures for all CpGs that allow a direct update of methylation rates—the measure of interest—and thus avoids expensive hard disk writes of intermediate alignment files. Using this index, FAME aligns a read in three phases, (i) MCpG candidate retrieval, (ii) verification of candidate MCpGs, and (iii) methylation calling (Fig. 2b). In the following, we explain the details of the index and how to use it to align a read and call methylation rates.

### 2.2 Index

For index construction, a given reference genome is segmented into small windows of about 2 kb length, called Meta CpGs (MCpGs). Each MCpG stores the set of CpGs it comprises and their respective genomic location. Every second *k*-mer within each MCpG is indexed using a fast rolling hash function from the library ntHash (Mohamadi *et al.* 2016), replacing all Cs by Ts in each spaced *k*-mer. All false positives introduced by this replacement are later resolved in the alignment phase. Such spaced *k*-mers are substrings of length k that are masked with a spaced seed that consists of *k* positions that are either the original letter or a wildcard. A spaced *k*-mer thus matches a length *k* genomic sequence if for each position the letter in the *k*-mer and genomic sequence are equal, or the *k*-mer contains a wildcard (see Supplementary Fig. S2). The hash values for spaced *k*-mers present in the reference genome, which are computed by ntHash, are used to populate a large hash table (the hash table implementation is a fast open source implementation of the Hopscotch hashmap https://github.com/Tessil/hopscotch-map), mapping spaced *k*-mers to the MetaCpGs they appear in.

To make the index more efficient, three optimizations are applied to the index. The first optimization, which is lossless, removes all except one *k*-mer object belonging to the same MCpG within a hash table cell (i.e. *k*-mers that have the same hash value), as this information is redundant. The second optimization throws out a distinct *k*-mer that occurs more than a predefined threshold, as such *k*-mers are likely to belong to repetitive regions. Repetitive genomic regions are problematic during an alignment, as reads stemming from such regions can map to all repeats and hence no unique match can be found. Removing highly repetitive *k*-mers from the index prevents matching to those repeats, thus avoiding a costly but unsuccessful alignment phase. This further reduces the index size and hence memory requirements. One downside of this approach is that low-quality reads stemming from the flank of a repetitive region might not be matched, as for such reads there might be too few *k*-mer hits—due to the repetitive *k*-mer filter and errors in the remaining *k*-mers—for the correct MCpG to be found. The third optimization introduces a bitmask for all reference *k*-mers. This bitmask has a 1 for all Ts in the unconverted refence *k*-mer, i.e. actual Ts in the reference, 0 for all other position in that reference *k*-mer. For a given read *k*-mer, we can thus discard reference *k*-mers if the read *k*-mer has a C at any positions with a 1 in the corresponding reference bitmask. This allows to reduce the number of false positives by a quick bitmask check already in the candidate retrieval phase.

With the index structure at hand, we can now efficiently retrieve candidate regions for bisulfite-aware alignment, which is explained in the following section.

### 2.3 Alignment

To align a read to the reference, FAME runs trough three phases (compare Fig. 2b). In the first phase, the *candidate retrieval*, each spaced *k*-mer of the read is looked up in the index using its hash value provided by ntHash. For each *k*-mer we, hence, get a collection of MCpGs where this *k*-mer might occur in. This way we obtain for each read an upper bound on how many of its *k*-mers are present in each MCpG. By setting a minimal number of *k*-mer occurrences *q* in a MCpG, most MCpGs can be excluded from further alignment steps.

In the second phase, similar to previous work (Otto *et al.* 2012), we employ a bisulfite-aware bitvector algorithm to solve the asymmetric mapping problem using edit distance and verify read matches to all candidate windows. In particular, FAME builds a Shift-And automaton for the read (Baeza-Yates and Gonnet 1992), but initializing the bitmask for Cs to all positions where a C **or a T** is present in the read. An example of a bisulfite aware Shift-And automaton is given in Fig. 1c, which can be easily generalized to an approximate Shift-And automaton (Supplementary Fig. S3). All candidate MCpGs are queried to the automaton to find true matches for a given read.

In the third phase, the unique best hit—if one exists—is used to compute an alignment between the read and the original reference genome at the matching position retrieved from the second phase. Instead of writing out intermediate alignment files, the CpGm counts are updated immediately in the CpG index. FAME applies a Dynamic Programming computation of the Levenshtein distance to find the best alignment between read and the matching subsequence of the reference leveraging banded alignment matrices, with the band width given by the number of errors in the unique best hit.

For paired end reads the three phases work analogously, but are applied to both reads enforcing constraints on the matching corresponding to the insert size. We first generate candidate MCpGs for each of the two reads. An additional pruning step can then be applied after the first phase by throwing out all MCpGs of one read that do not have a corresponding MCpG that lies within insert size distance away to the paired read. For our usual 2 kb MCpG windows that means only considering an MCpG of read 1, for which either that same MCpG or one of the adjacent MCpGs are present for read 2, and vice versa. For the second phase, we match all MCpGs of each read and check if there is a unique best match, considering all combinations of match pairs between read 1 and read 2 that are at most insert size bps apart looking at the genomic coordinates of the match.

Storing all data in memory allows highly parallel implementation of all three steps and results in fast processing, while the memory consumption is similar to other methods, requiring less memory than Bismark or Segemehl (see Supplementary Table S1). Further, there is no need for large disk storage capacities for intermediate files, a bottleneck imposed by other methods, which generate up to terabytes of data for a single single-cell dataset.

All alignment phases can naturally be extended to support single cell experiments (compare Fig. 2b). For that, reads are processed as batches, where each batch represents the read yield of a single cell. Each of these batches runs through the three alignment phases of FAME and methylation rates are estimated per batch and streamed into a file. This results in a matrix containing all single cell experiments as rows, avoiding any overhead caused by repeated loading of the index for each cell, or excessive I/O caused by writing out methylation calls for all CpGs for each individual cell, which usually have sparse coverage.

## 3 Materials and methods

In the following, we discuss data generation and preprocessing along with performance metrics used for our evaluation and comparison.

### 3.1 Synthetic data preparation

To carry out an evaluation on data with known ground truth, we generate synthetic data resembling high quality WGBS reads. Each synthetic dataset consists of 25 million 100 bp reads sampled uniformly from chromosome 22 of human reference genome hg19. For each CpG in the genome we flip a coin and draw a methylation rate *p* either from N(0.2, 0.08) or N(0.8, 0.08), reflecting hypo- or hypermethylated CpGs by Gaussians with low or high mean, respectively, as usually observed in bulk sequencing data. For each generated read, we methylate each CpG in the read with probability *P* given by the reference CpG. Conversion success rate is set to 99%, which is commonly observed in real WGBS experiments. Cytosines in non-CpG context are methylated with probability 0.01 reflecting real mammalian genomes. Finally, we introduced *l* errors, where *l* was drawn from a Poisson with λ=0.5 to simulate base calling errors. For paired end reads we generated 25 million pairs of two reads generated as explained above, with the restriction that reads should only be between 100 and 400 bp apart from each other, drawn uniformly at random, reflecting the allowed insert size of paired end protocols. As we know the ground truth mapping positions of each read and the methylation counts for each CpG, we can compute the Root Mean Squared Error (RMSE) and Spearman correlation coefficient using all CpGm values. We considered all CpGs, where at least one tool mapped more than five reads. We measured the runtime as the sum of time by alignment and by methylation calling.

### 3.2 WGBS and EPIC bead data processing

In preparation for the comparison on real data, we downloaded the 5 WGBS paired-read datasets of LNCaP cell by Pidsley *et al.* (2016), along with corresponding EPIC bead methylation calls. We then processed the EPIC bead data using RnBeads (Assenov *et al.* 2014, Müller *et al.* 2019) to obtain methylation rates for all CpG positions on the EPIC bead array. For the two replicates, we averaged the methylation rates per CpG. The 5 WGBS read sets were pooled, leading to about 437 million reads. Adapters were trimmed using trim galore (http://www.bioinformatics.babraham.ac.uk/projects/trim_galore/), following the original approach by Pidsley *et al.* (2016):

trim_galore —length 85 —max_n 1 —paired —gzip

 LNCaP_pooled_1.fastq . gz LNCaP_pooled_2.fastq . gz —o trimmed/

For the evaluation, we considered the 841 708 CpG positions out of 843 385 CpGs present on the array, where at least one tool mapped more than 5 WGBS reads. We considered the EPIC derived CpGm values as a baseline for this comparison.

### 3.3 Single-cell WGBS data processing

To test available methods on a recent single-cell dataset, we consider data of induced pluripotent stem cells generated in a study by (Linker *et al.* 2019). Analogue to the original study, we preprocessed the single cell data using trim-galore.

trim_galore —paired —clip_R1 6 —clip_R2 6 —gzip —length 90

    CELLID_1. fastq . gz CELLID_2. fastq . gz

### 3.4 Performance metrics

In previous work, the number of aligned reads has often been considered a good proxy for mapper performance. However, this measure is misleading as reads aligned to the wrong position are counted positively towards performance. As an extreme example, consider a mapper that maps all reads to the first position of chromosome 1, resulting in 100% aligned reads, which is of course a wrong alignment. Thus a better approach is to report the number of correctly mapped reads, doing an extensive postprocessing of alignments to compare to known ground truth read positions on synthetic datasets. For real data, such a ground truth is impossible to obtain.

With the actual goal of quantifying DNA methylation, we can mitigate this issue by evaluating how close the predicted methylation rate is to the actual methylation rate. Hence, the performance is good if and only if the alignment is good (i.e. correct). We can leverage orthogonal experiments measuring CpG methylation on real data, such as EPIC arrays, to obtain alignment independent methylation rates against which we can evaluate all WGBS estimates. We measure predicted against actual methylation rate in terms of the Root Mean Squared Error (RMSE): where *C* denotes the set of all CpGs with measurements and |C| denotes the size of this set. $m_{c}$ denotes the ground truth (simulations or EPIC array) CpGm value of CpG *c* and ${\hat{m}}_{c}$ denotes the estimated value of one of the investigated tools based on WGBS data. Similarly, Spearman rank-based correlation was computed to measure the agreement between baseline CpGm values and estimates from the compared tools.


$$\mathrm{RMSE}=\sqrt{\frac{\sum_{c\in C}{\left(m_{c}-\hat{m_{c}}\right)}^{2}}{\left|C\right|}},$$

We consider all CpG sites where at least one tool mapped more than 5 reads (considering this CpG as mappable) and set the predicted methylation rate of tools that do not have any mapped reads at this CpG to 0 if the ground truth methylation rate is larger than 0.5, and to 1 if the ground truth methylation rate is smaller than 0.5. This introduces a small penalty if a tool is not able to cover a region during alignment, although it was possible.

## 4 Results

We have developed FAME as a novel method for the quantification of DNA methylation rates at CpGs directly from bisulfite read data without producing intermediate alignment files (Fig. 2). We compare FAME against existing RA and BA mapping approaches in terms of runtime and quality of called methylation rates both on synthetic and real data. We note that there are a plethora of tools available and we have selected a competitive subset of those for our comparison, which are the most state-of-the-art algorithms that have full capabilities for alignment and methylation calling (more details, including why certain tools are excluded, in Supplementary Table S2). The competing RA mappers were BratNova (Harris *et al.* 2016), Bismark (Krueger and Andrews 2011), and gemBS (Merkel *et al.* 2019), the competing BA mappers were BSmap (Xi and Li 2009), and Segemehl (Otto *et al.* 2012) (method parameters in Supplementary Section S5).

We measure the quality of called methylation rates in terms of Spearman correlation coefficient and the RMSE between predicted and baseline methylation rates. With ground truth methylation rates known for synthetic data, and methylation estimates available from orthogonal experiments for real data, we can thus evaluate each method’s ability to call methylation rates. Furthermore, methylation calls serve as a proxy for read alignment performance, as the methylation calls are good if and only if the alignments are good. Thus, in our evaluation we are not concerned with the issues of typical read alignment evaluations, where ground truth read positions are inherently hard to obtain for real data.

### 4.1 Hyperparameter optimization

**Figure 3.**
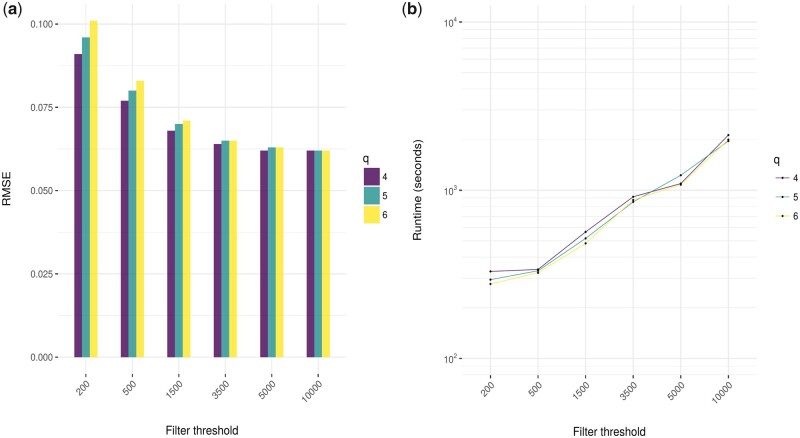
Results for different index parameters. Depicted are the results of a grid search for varying *q* (color), and filter threshold *t* (*x*-axis). (a) Root Mean Squared Error (RMSE, smaller is better) compared between actual and predicted methylation rates on the synthetic grid dataset and (b) runtime for the same.

We first designed a simulation study to determine FAME’s default parameters on an independent synthetic dataset following the *in silico* data generation described in Section 3. The results for varying filter threshold *t—*the maximum times a *k*-mer is allowed to occur in the genome to be considered for the index—and the minimum number of reads *q* for a MCpG to be considered for alignment are given in Fig. 3. As expected, we can see that with larger *t* and smaller *q* the performance worsens but the runtime improves. These parameters provide a natural tradeoff between accuracy and runtime. We settle for default values t=1500, q=5, as they show a good balance between runtime and prediction accuracy and use them throughout the rest of the paper.

### 4.2 DNA methylation calling on synthetic read set

**Figure 4.**
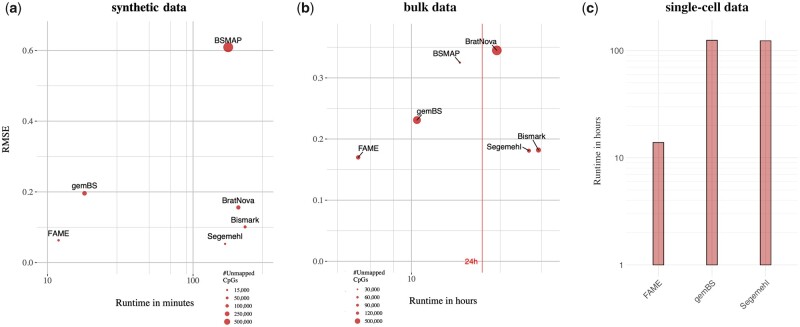
Comparison on bisulfite sequencing data. Visualization of results for simulated bulk sequencing WGBS reads (a) and real WGBS data (b) of LNCaP cell lines (Pidsley *et al.* 2016). We compare runtime (*x*-axis) against error of the predicted methylation values as RMSE (*y*-axis). The size of each point indicates the number of unmapped CpGs. In case of the real world data, EPIC arrays serve as baseline methylation calls against which we compare the methods. Runtimes in hours on log-scale for scBS-seq (c) of 192 cells (Linker *et al.* 2019).

Next, we generated synthetic data (25 million reads) sampled from human chromosome 22 resembling WGBS sequencing data (see Section 3). The performance of each tool was measured in terms of the combined runtime of read alignment and methylation calling on the whole genome, as well as quality of methylation calls. The results are given in Table 1 and Fig. 4a, a more detailed visualization of the called methylation rates can be found in Supplementary Fig. S4.

**Table 1.** Method comparison.^a^

Metrics		Reduced alphabet mapper			Bisulfite aware mapper		
		Bismark	BratNova	gemBS	BSmap	FAME	Segemehl
Synth. data 25M							
Runtime (h:min:s)		3:47:53	3:24:38	0:17:57	2:54:22	0:11:54	2:46:03
Performance							
RMSE		0.101	0.156	0.196	0.609	0.063	0.053
Spearman		0.93	0.86	0.78	–0.34	0.97	0.98
#missed CpGs		12 371	44 800	57 319	583 528	5130	4444
Real data 437M							
Runtime	(h:min:s)	48:13:18	28:46:20	10:41:38	18:12:25	5:09:46	42:50:24
Performance							
RMSE		0.182	0.345	0.231	0.325	0.17	0.181
Spearman		0.86	0.61	0.8	0.79	0.88	0.86
#missed CpGs		132 704	747 904	442 318	28 208	88 470	71 571

a All methods are compared on synthetic (top) and real WGBS data (bottom). Runtime of alignment and methylation calling (32 threads) and accuracy given as Root Mean Squared Error (RMSE, lower is better) and Spearman correlation (higher is better) are reported. The number of CpGs for which no reads were aligned (#missed CpGs) is given in a separate row.

We observe that FAME yields accurate results, improving on all tested RA mappers and yielding as good results as the most accurate mapper Segemehl. More importantly, FAME is running an order of magnitude faster than Segemehl and Bismark, the most accurate RA and BA approaches in our test, bringing down the computation time from several hours to minutes, whereas the second fastest method gemBS showed poor methylation rate calls.

Our results further confirm that BA mappers, such as FAME and Segemehl, yield more accurate methylation rates than RA mappers, which supports the intuition considering the false positive matchings introduced by RA approaches (see Fig. 4a).

### 4.3 Predicting DNA methylation on real world data against EPIC bead

In the next experiment, we used real WGBS data (437 million paired reads) from the LNCaP cell line, which were accompanied by EPIC array measurements (Pidsley *et al.* 2016). EPIC arrays provide an alternative large-scale measurement of CpGm values, which is not concerned with the same biases as WGBS.

The results, visualized in Fig. 4b and Table 1, show that FAME is 8, 9.5, and 2 times faster than Segemehl, Bismark, and gemBS, respectively. In terms of accuracy, FAME outperforms all RA and BA mappers on this real dataset (Fig. 4b, Table 1, Supplementary Fig. S5). FAME thus provides a unique balance between WGBS processing speed and accuracy. Further analyses showed that Segemehl and FAME are on par with the highest number of covered CpGs, while all other methods show up to an order of magnitude more CpGs with no aligned read. As with the synthetic data experiment, this real dataset highlights inferior RA mapper performance compared to BA mappers, which has been independently reported in the literature (Frith *et al.* 2012, Otto *et al.* 2012).

The synthetic datasets used to evaluate the methods and the processed EPIC bead data are deposited online for reproducibility (see Data Availability statement).

### 4.4 Scaling to single cell data

To this end, the analysis showed that FAME yields accurate results in a fraction of time of the state-of-the-art. The following analysis is concerned with the scalability of methylation calling to the sequencing yields produced from single cell experiments. A unique feature of FAME is to directly support single cell WGBS datasets, where reads of each cell are aligned, counted, and methylation rates are put out per cell, without additional overhead. We thus investigated the performance on single cell data using a dataset of 192 cells from Linker *et al.* (2019). As processing this data is time-intensive, we used gemBS and Segemehl to represent the fastest, respectively most accurate, competing method from our previous analyses and applied them on this single cell dataset. FAME was able to process all cells within 11 h, which is an order of magnitude faster than competitors, which both took more than 5 days to finish the analysis (see Fig. 4c). All runtimes were achieved while parallelizing on 32 cores on a modern server and demonstrate that single cell data methylation calling is a huge bottleneck, even more with the recent advances of combinatorial indexing and other platforms that yield sequencing of thousands of cells per run (Vitak *et al.* 2017, Mulqueen *et al.* 2018).

## 5 Discussion and conclusion

In the scope of this work, we present a novel method to efficiently quantify CpG methylation from WGBS data and analyse its ability to accurately predict methylation rates on synthetic and real world data in comparison to state-of-the-art methylation callers.

To overcome limitations of evaluations that are solely based on mapping efficiency, here we directly evaluate on synthetic data how close the predicted methylation rates are to the ground truth. Thus, any inefficiencies in read mapping, wrongly mapped reads, or mapping biases are captured by this evaluation as they lead to wrong or biased methylation estimates, which otherwise could be missed. While we generated the synthetic data in close resemblance of how reads are actually produced by a sequencing protocol, there might be issues in real world data that are not captured by this dataset. We, hence, compared on real-world data with the same underlying idea, to leverage baseline methylation rates to evaluate the estimation capabilities of the methods at hand. The actual methylation rates of a cell are unknown, yet we can do our best to evaluate on real data taking into account orthogonal experiments of quantification of DNA methylation. Here, we leverage estimates from EPIC arrays as comparison to the WGBS calls of each tool. While not being an actual ground truth, it gives a different view on methylation rates that does not suffer from the issues inherent in the process of read alignments with asymmetric mappings (Pidsley *et al.* 2016). In our analysis we settled for the EPIC bead data, as it is to the best of our knowledge the most reliable and state-of-the-art protocol for methylation analysis across the full genome besides WGBS.

Overall, both synthetic and real world evaluation matched our expectations that bisulfite aware mapper yield more accurate methylation estimates than tools that use a reduced alphabet for mapping, which is in line with previous findings (Frith *et al.* 2012, Otto *et al.* 2012). The state-of-the-art bisulfite aware mapper Segemehl yields consistently highly accurate results, but is prohibitively slow in comparison to other tools. The widely used tool Bismark also showed consistently reasonable performance despite being a reduced alphabet mapper, yet is less accurate than Segemehl or FAME. To our surprise, the method gemBS showed poor estimation quality in our tests. Similar issues of gemBS have been reported independently in a recent comparison study on plant data (Grehl *et al.* 2020). Our method FAME shows among the best performance regarding estimation quality, while being an order of magnitude faster than the state-of-the-art. Especially for recent large-scale single cell sequencing datasets, this eliminates the need for high performance computing capabilities, which opens the alignment and methylation calling of WGBS single cell datasets to a broader community.

While FAME heavily relies on the special index structure to achieve these fast and accurate estimates, its memory requirement is comparable to gemBS, and less than Bismark and Segemehl (Supplementary Table S1). Yet, all of these methods, including FAME, have memory requirements that still need a machine with 40 GB of memory for human WGBS data. Reducing the index size, e.g. by using further compression techniques or sparsification of *k*-mers, would be an interesting line of work to be able to deploy FAME on standard laptops.

Methylation calling is arguably the major application of WGBS alignment methods, yet there are interesting downstream applications such as epiallele calling or cellular deconvolution that require WGBS read alignment information. FAME sacrifices writing out alignment files to increase on speed and reduce hard disk footprint compared to competitive tools, which currently renders FAME inapplicable to those downstream applications that require read-level methylation estimates. As FAME computes an alignment, it would be in principle possible to produce alignment files, but at the cost of breaking highly parallelized algorithmic steps using the index structure, adding runtime for costly IO file writing operations. Hence, further developments would be necessary to allow efficient alignment file generation for these downstream tasks.

The introduced conceptual change to estimating CpG methylation without intermediate WGBS alignment files is paralleled by earlier development of methods for expression quantification from RNA-seq data (Patro *et al.* 2014, Bray *et al.* 2016, Patro *et al.* 2017). However, these methods do not compute a complete semi-global alignment for an individual read as basepair-level information is not required for the quantification process, but instead only use exact k-mer counts to perform a so-called pseudo-alignment.

The focus of our work lies on the estimation of CpG methylation from WGBS data. As such, it is particularly well suited for the analysis of mammalian genomes. While extending to methylation in arbitrary context, e.g. for applications in plant biology, is generally possible, our constructed index would be much larger, forgoing the advantage in speed that our compact index structure provided in this work. For a specific methylation context, such as GpC methylation, our algorithm can easily be transferred. An interesting avenue for future work would thus be an adaption of our algorithm to NOME data by building the index for GpCs instead of CpGs. Hence, we could use FAME to get open chromatin calls from NOME-seq experiments (Rhie *et al.* 2018), leveraging FAME’s accuracy and speed for a different data modality. Similarly, adaptations of our index and algorithm to accommodate SLAM-seq based experiments (Muhar *et al.* 2018) would make for exciting future work. In both application scenarios, NOME-seq and SLAM-seq, correctly resolving an asymmetric mapping, introduced by a base conversion in the protocol, is crucial to obtain accurate results and suitable methods are needed for fast processing for these new generation of techniques.

## Supplementary Material

btad386_Supplementary_Data

## Data Availability

Our generated synthetic data of our performance analysis is publicly available through https://zenodo.org/record/2574694. The bulk WGBS data by Pidsley *et al.* (2016) was deposited through accession IDs SRR4238609 up to SRR4238613 in the Sequence Read Archive (SRA) by the authors of that study. Linker *et al.* (2019) published their data through project ID PRJEB15062 through the European Nucleotide Archive (https://www.ebi.ac.uk/ena/browser/view/PRJEB15062), the raw reads of the methylation analysis forming the basis for our experiments.
